# Tidal and hydrological periodicities of seismicity reveal new risk scenarios at Campi Flegrei caldera

**DOI:** 10.1038/s41598-018-31760-4

**Published:** 2018-09-14

**Authors:** Simona Petrosino, Paola Cusano, Paolo Madonia

**Affiliations:** 1 Istituto Nazionale di Geofisica e Vulcanologia, Sezione di Napoli - Osservatorio Vesuviano, via Diocleziano 328, 80124 Napoli, Italy; 2 Istituto Nazionale di Geofisica e Vulcanologia, Sezione di Palermo, via Ugo La Malfa 153, 90146 Palermo, Italy

## Abstract

The volcano-tectonic seismicity occurring at Campi Flegrei caldera during its present unrest phase, started in 2005, is distributed into time-clustered events emerging from a background composed of earthquakes with higher inter-arrival times. Here, we show that clustered seismicity is cyclically recurrent at time scales from semidiurnal to annual, matching tidal and hydrological periodicities. These results suggest that volcano-tectonic seismicity at Campi Flegrei caldera is driven by both variations in the deep magmatic feeding system and exogenous phenomena, as rainfall or global inflation/deflation cycles of the Earth’s crust, controlled by the lunisolar interaction. Consequently, the role of exogenous triggers in the evolution of the present unrest phase should be properly considered in the elaboration of volcanic risk scenarios, presently limited to the study of surface indicators of deep phenomena.

## Introduction

External forcing of seismic and volcano-seismic activity by hydromechanical coupling of instable, shallow fault and hydro-magmatic systems with infiltrating rainwater has been documented in various geological settings^1,2^, as well as synchronization between tides and hydrothermal seismicity^3–6^. However, the possible effects of exogenous processes in the evolution of a restless volcano from unrest to eruptive conditions do not commonly deserve the necessary attention, which is instead focused on the interpretation of the surficial manifestations of deep processes. This approach could be critical when dealing with restless calderas, whose eruptions can be preceded only by small unrest signals^7,8^.

**Figure 1:**
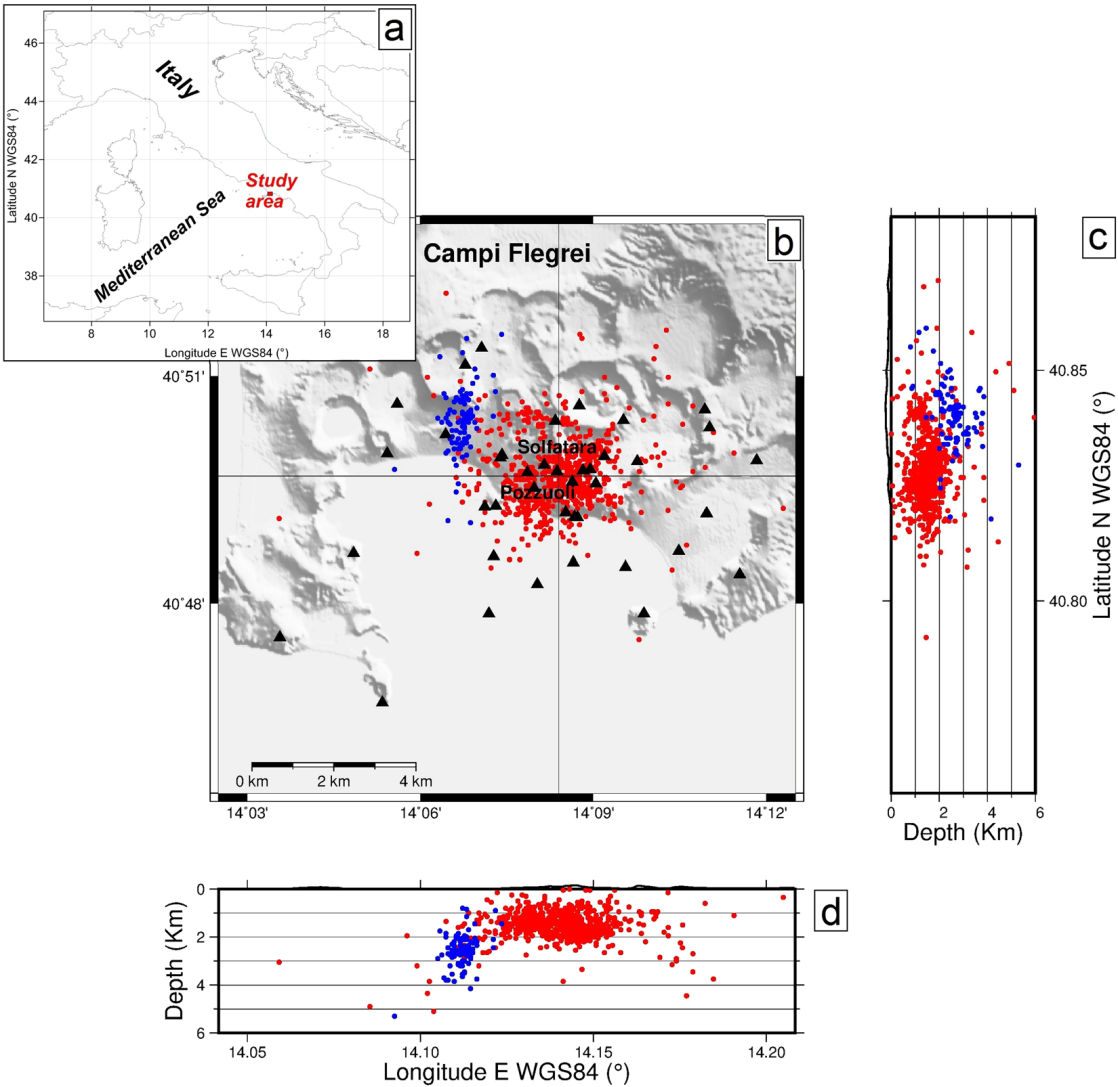
Location of the study area (**a**) and VT events (red and blue dots) at the CFc: (**b**) map, (**c**) N-S and (**d**) E-W depth profiles. The depth is referred to sea level. Blue dots represent the September 2012 seismic swarm. Triangles are the seismic stations of the INGV-OV network.

Due to its location inside the densely inhabited conurbation of Napoli, the Campi Flegrei caldera (CFc) is one of the most hazardous active volcanic systems of the world. Since at least the Roman age it has been affected by a peculiar ground deformation phenomenon, called “bradyseism”, consisting of alternating, recurring phases of slow inflation and deflation, whose maximum amplitudes have been recorded close to the city of Pozzuoli^9^. Ground deformation is associated with volcano-tectonic (VT) and long-period (LP) seismicity, diffuse soil CO_2_ degassing and a strong fumarolic activity, mainly concentrated at La Solfatara and its immediate surroundings (Fig. 1)^9^.

**Figure 2:**
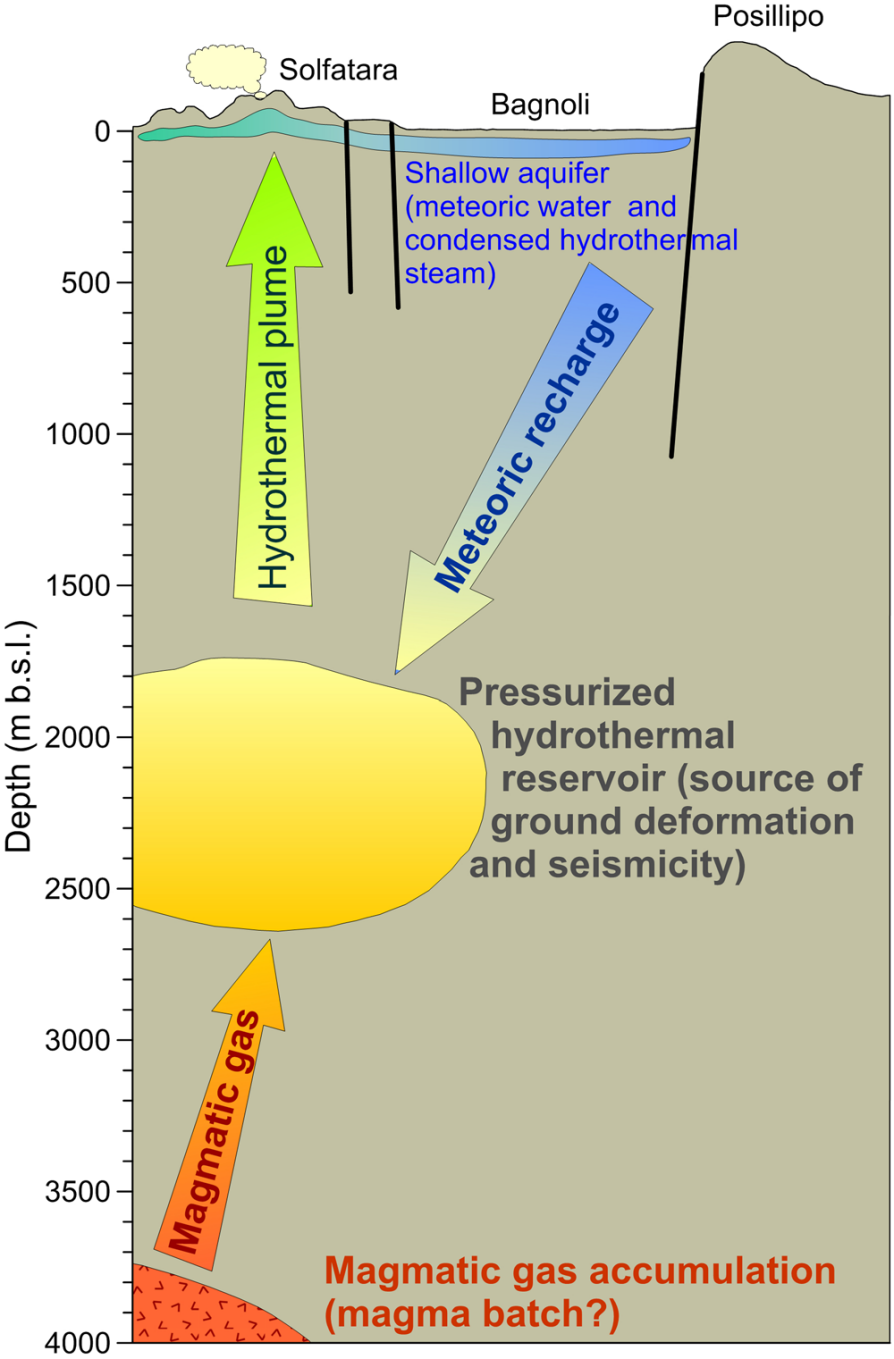
Schematic representation of the CFc geofluid circulation system (modified from literature data^9,10^).

The geodynamic engine of the bradyseismic movements is supposed to be a multi-stage magmatic-hydrothermal system (Fig. 2) composed of^10^: (i) a circa 4 km deep gas accumulation zone, maybe related to a small magma batch^11^, releasing hot gases toward (ii) a circa 2 km deep hydrothermal reservoir, where upwelling magmatic fluids mix and vaporize meteoric water, generating ground deformation, seismicity and (iii) a hydrothermal gas plume, supplying the shallow degassing system of La Solfatara and its surroundings, which releases up to 2000 t d^−1^ of CO_2_.

After 20 years of prevailing deflation, since 2005 the CFc has experienced a new uplift phase, with a total vertical displacement of 40 cm measured at the end of 2016. The vertical movement has been not constant in time, alternating periods of increased uplift rate with intervals of subsidence or stationary conditions. A strongly accelerating ground uplift rate was recorded between April 2012 and January 2013, inducing the Italian Civil Protection to raise the volcanic alert level from “background” to “attention”^12^.

This episode was accompanied by an anomalous seismic swarm, occurred on September 7^th^ 2012 and lasted about 1.5 hours, consisting of about 200 earthquakes with hypocentres located outside the area normally affected by seismicity (Fig. 1) and very close to the northern edge of the inferred inflating magmatic reservoir^12^. The sequence was associated with fracturing along pre-existing faults, triggered by the volumetric increase of the magmatic body beneath the CFc^13^. Moreover, a good correlation among ground deformation, seismicity and geochemical characters of surface hydrothermal degassing was found, and it was interpreted as the effect of recurrent injections of magmatic fluids into the 2 km-deep hydrothermal reservoir^10,12,13^.

It is worth nothing that, even though the meteoric water input is a fundamental component of the 2-km deep hydrothermal reservoir (Fig. 2), the whole studies on the CFc most recent unrest have been focused on the search of proxies of magma and deep fluid injections. On the other hand, hydrological cycle variations or solid Earth tides have scarcely been investigated as a possible concurrent cause of CFc unrests. Actually, low-frequency signals generated by the activity of the shallow geothermal reservoir, and modulated by a tidal contribution on the diurnal/semidiurnal time scale, have been detected in the seismic noise recorded during 2006–2010 at the CFc^4^.

Following these considerations, we analyse and compare time series of Earth tides, seismicity, rainfall, atmospheric pressure and ground deformation at the CFc in the time span 2005–2016, looking for possible tidal and hydrological triggers of hydrothermal activity and its related seismicity.

## Results

### Statistical analysis of volcano-tectonic earthquakes compared to periodicity of Earth tides, rainfall and atmospheric pressure

The CFc uplift episodes have been accompanied by VT seismicity often occurring in swarms; these earthquakes generally concentrates in few hours or even minutes and they are usually located beneath the Solfatara-Pozzuoli area, at depth up to 4 km^14,15^ (Fig. 1). Chiodini *et al*.^16^ have shown that the post-2000 seismicity is composed by events with high inter-arrival times (>3days, considering the mode of the distribution) and low inter-arrival time (<15 minutes) population corresponding to swarms.

**Figure 3:**
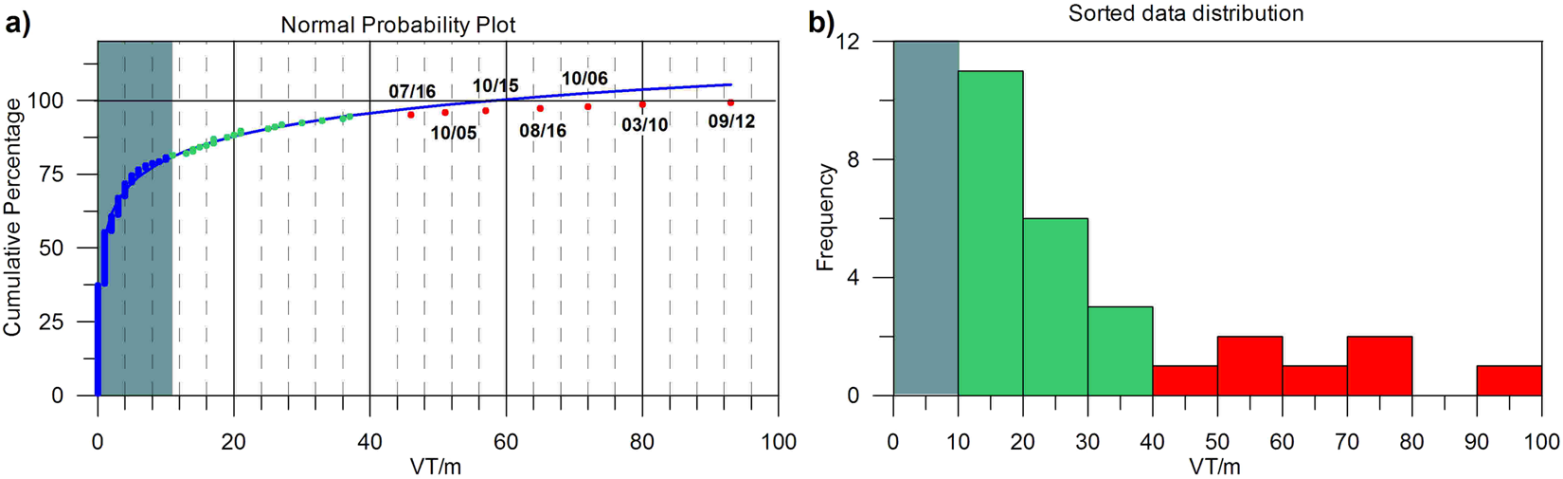
(**a**) Normal probability plot with cumulative percentage of sorted earthquakes (VT m^−1^, blue/green dots), logarithmic fit of the probability (blue line), and data with the highest RMS fit-values (red dots). (**b**) Sorted data (VT m^−1^) distribution. The green areas delimit no-significant data.

We analysed the VT seismicity occurred at Campi Flegrei (details in the Method section) during the years 2005–2016 and recorded by the seismic network of Istituto Nazionale di Geofisica e Vulcanologia – Osservatorio Vesuviano (INGV – OV). We selected earthquakes of duration magnitude greater than −1.0, which is the estimated magnitude of completeness (earthquake catalogue included as supplementary material). We applied the Normal Probability Plot technique^17,18^ (see Method section) to the time series of VT monthly rate to distinguish among different population of data, as sparse background events or clustered seismicity. The cumulative frequency of the number of VTs per month (N = VT m^−1^) was calculated and the Root Mean Square (RMS) parameter was used as indicator of the fit goodness. A natural logarithmic function best reproduces the data distribution reflecting a lognormal distribution (Fig. 3a). For this fit, the minimum RMS level is reached at Nmin = 10 VT m^−1^ and it remains almost constant until Nmax = 37 VT m^−1^. The inspection of the probability histogram (Fig. 3b) suggests the presence of seven outliers, laying in the portion of the curve that begins to deviate from the lognormal distribution: 46 VT m^−1^ on July 2016, 51 VT m^−1^ on October 2005, 57 on October 2015, 65 on August 2016, 72 on October 2006, 80 on March 2010 and 93 on September 2012. Hereinafter, seismicity deviating from the lognormal distribution (N > Nmax) will be referred to as “Outlier Seismicity” (OTS). According to the threshold of Nmin = 10 VT m^1^ estimated by the previous analysis, we separated our dataset into two subsets: the first one contains the VTs below Nmin (hereinafter referred to as “Background Seismicity”, BGS), while the second one corresponds to the distribution above Nmin. This last subset contains the most part of the seismic swarms occurred at the CFc and we will refer to it as “Clustered Seimicity”, CLS. We remark that according to this definition, CLS is not synonymous of seismic swarm.

**Figure 4:**
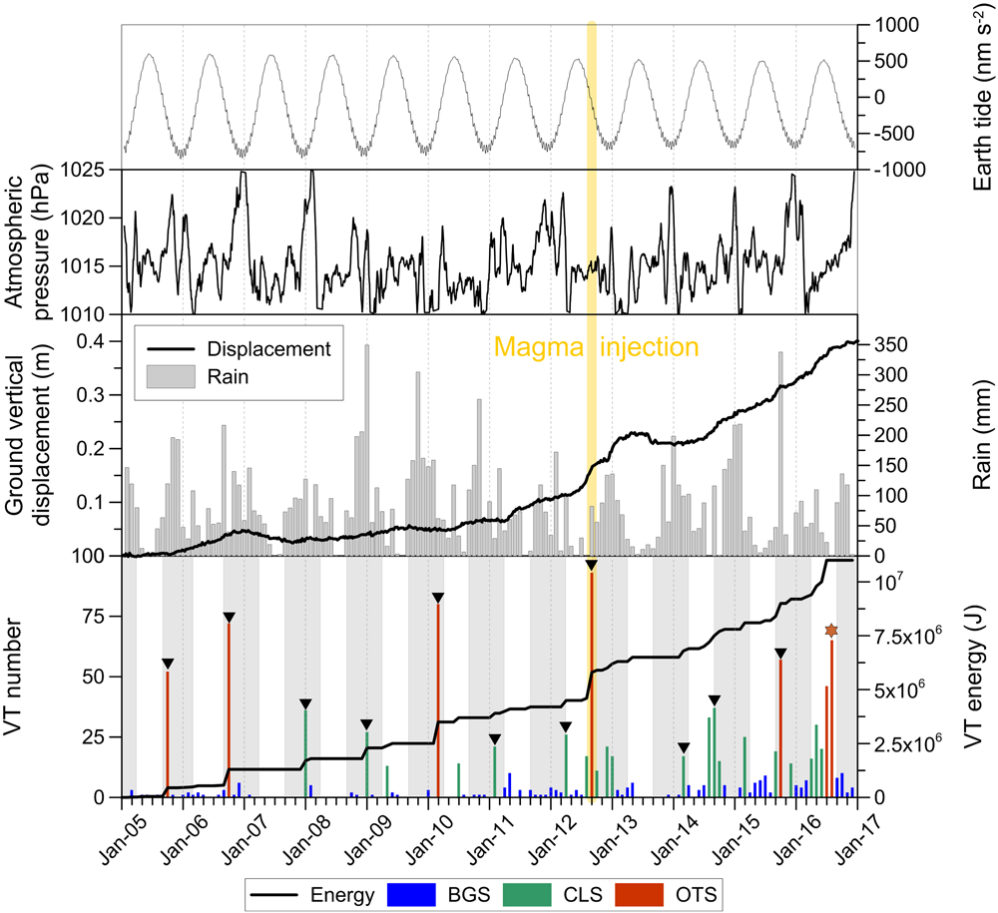
Solid Earth tides, atmospheric pressure, rainfall, ground uplift, VT number and cumulated energy. VT earthquakes subdivided in BGS, CLS and OTS according to Fig. 3. The most numerous swarms of each hydrological year occurred during the wet season (grey bands) are represented with black triangles, while the only one falling in a dry season is marked by the red star.

The following analytical step was focused on the search for possible interferences between the seasonal periodicity of atmospheric parameters, e.g. rainfall amount and atmospheric pressure, and occurrence of seismicity. Monthly values of VT number, rainfall amount and atmospheric pressure are illustrated in Fig. 4; we used different colours in the bar chart representing VT number for distinguishing among BGS, CLS and OTS. In the same graph we also reported the cumulative VT seismic energy, calculated by applying the Gutenberg-Richter relationship for the Campi Flegrei area^19,20^:

1 $${\rm{LogE}}=9.9+1.9\mathrm{MD};$$

where E is the energy (in joules) and MD is the duration magnitude. Looking at the temporal distribution of the VTs (Fig. 4), it is evident the periodic occurrence of the seismic activity: the earthquakes generally gathered in swarms concentrated in particular time intervals. Until 2012 intense swarms are sparse and alternated to phases of very low seismicity. The 2012, characterized by one of the most intense swarms, is followed by a year (2013) with scarce seismic activity. Finally, since 2014 VT swarms have been more frequent, although they are composed of a relatively lower number of earthquakes compared to the previous years. This particular behaviour is also reflected in the cumulative distribution of the energy, which is more discontinuous in 2005–2012 and smoother in 2014–2016. The September 2012 swarm also represents an evident dividing line between two different seasonal distributions of VT seismicity.

Based on the average intra-annual distribution of rainfall (see Method section for related details), the average hydrological year at the CFc starts on September and it is divided into two distinct seasons: the wet season (grey bands in the lower graph of Fig. 4), running from September to March, and the dry season, from April to August. Before the September 2012 swarm, VT events mainly occurred during the wet season, but after that swarm seismicity was more continuously distributed throughout the year, with slightly more frequent events during the dry months with respect to the wet season. Another relevant feature is the evident seasonality of OTS and annual relative maxima of CLS: with the sole exception of 2016, all the most numerous (and energetic) VT swarms occurred during the wet season, both before and after September 2012. The seasonal distribution of VT earthquakes and its time variations are better evidenced in numerical form in Table 1, where we report both the average monthly rainfall amount and VT number during the wet (September-March) and dry (April-August) hydrological seasons. VT number was calculated separately for BGS, CLS + OTS and BGS + CLS + OTS. We tested all the possible combinations, always excluding the September 2012 swarm and extending calculations to: (a) the whole observational interval 2005–2016; (b) the whole observational interval 2005–2016 excluding the year 2012 in the hypothesis that all the earthquakes occurred that year could represent a coherent seismic sequence, culminating in the September swarm; (c) the three sub-intervals 2005–2011, 2005–2012 and 2013–2016. Some interesting facts are evidenced by the values reported in Table 1. The seasonal distribution of rainfall is constant for all the possible time combinations, at the net of insignificant statistical fluctuations; moreover, including or excluding VTs occurred in 2012 do not substantially alter the results, so hereafter we will discuss all the cases including that year. BGS is homogeneously distributed throughout the year until the wet season starting from September 2012, while since 2013 a slightly higher number of events are observed during the dry months. A different behaviour is observed for both CLS + OTS and BGS + CLS + OTS, with much more events occurring in the wet season with respect the dry one for the 2005–2012 sub-interval. If the observational time span 2005–2016 is considered as a whole, no appreciable seasonal distributions are observed for BGS, while a higher frequency of VT events during the wet season is still evident for both CLS + OTS and BGS + CLS + OTS. Differently to the similitudes observed between VTs and rainfall periodicity, the atmospheric pressure signal is more variable (Fig. 4), with relative maxima generally falling in the winter season but significantly affected by inter-annual variations.

**Table 1:** Average monthly rainfall amount and VT number in the wet and dry hydrological seasons, calculated for the whole observational interval and for the pre- and post- September 2012 swarm sub-intervals, always excluding this swarm but both including and excluding the other events occurred in 2012. VT number was calculated separately for BGS, CLS + OTS and BGS + CLS + OTS (2012 included only because of the irrelevance of its exclusion, as shown by the data in the previous rows).

Time interval	Rain (September–March, mm m^−1^)	Rain (April–August, mm m^−1^)
2005–16	110.5	37.7
2005–12	119.8	40.4
2013–16	101.3	35.1
**Time interval**	**Earthquakes** **(September–March, VT m** ^**−1**^ **)**	**Earthquakes** **(April–August, VT m** ^**−1**^ **)**
*BGS*		
2005–16 inc 2012	1.24	1.37
2005–16 exc 2012	1.23	1.40
2005–11	0.82	0.80
2005–12	0.88	0.83
2013–16	1.96	2.45
*CLS* + *OTS*		
2005–16 inc 2012	6.88	4.40
2005–16 exc 2012	5.88	4.02
2005–11	5.14	0.77
2005–12	6.73	1.75
2013–16	7.18	9.70
*BGS* + *CLS* + *OTS*		
2005–16 inc 2012	8.56	6.03
2005–12	8.27	2.57
2013–16	9.14	12.95

**Figure 5:**
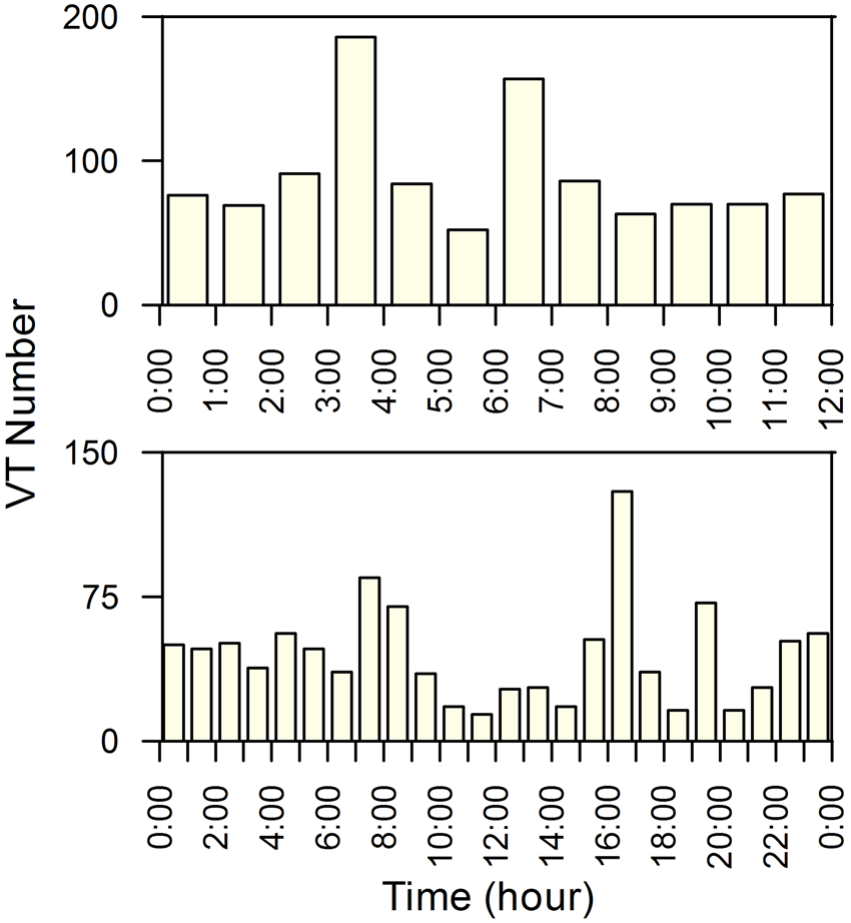
Hourly frequency distribution of the VT events from 2005 to 2016 during the 12- (upper panel) and 24-hour (lower panel) daily cycles.

In order to gain more insights into the periodical pattern of the earthquake occurrence we also considered its possible correlation with the solid Earth tides. First, we generated the theoretical tides^21^ at Campi Flegrei site for the time span 2005–2016 and compared them with the VT occurrence frequency, rainfall amount and atmospheric pressure (Fig. 4). As a general remark, it is noteworthy that Earth tides, rainfall amount and atmospheric pressure periodicities exhibit a constructive phase interference: relative maxima of rainfall and pressure and relative minima of Earth tides occur in late autumn-early winter. Conversely, minima of rainfall and pressure and maxima of Earth tides are recorded in late spring-early summer. In addition, periodicities at shorter time scales (diurnal and semidiurnal) of the seismic activity are also evident at a visual inspection of the VT occurrence frequency, as shown in Fig. 5.

**Figure 6:**
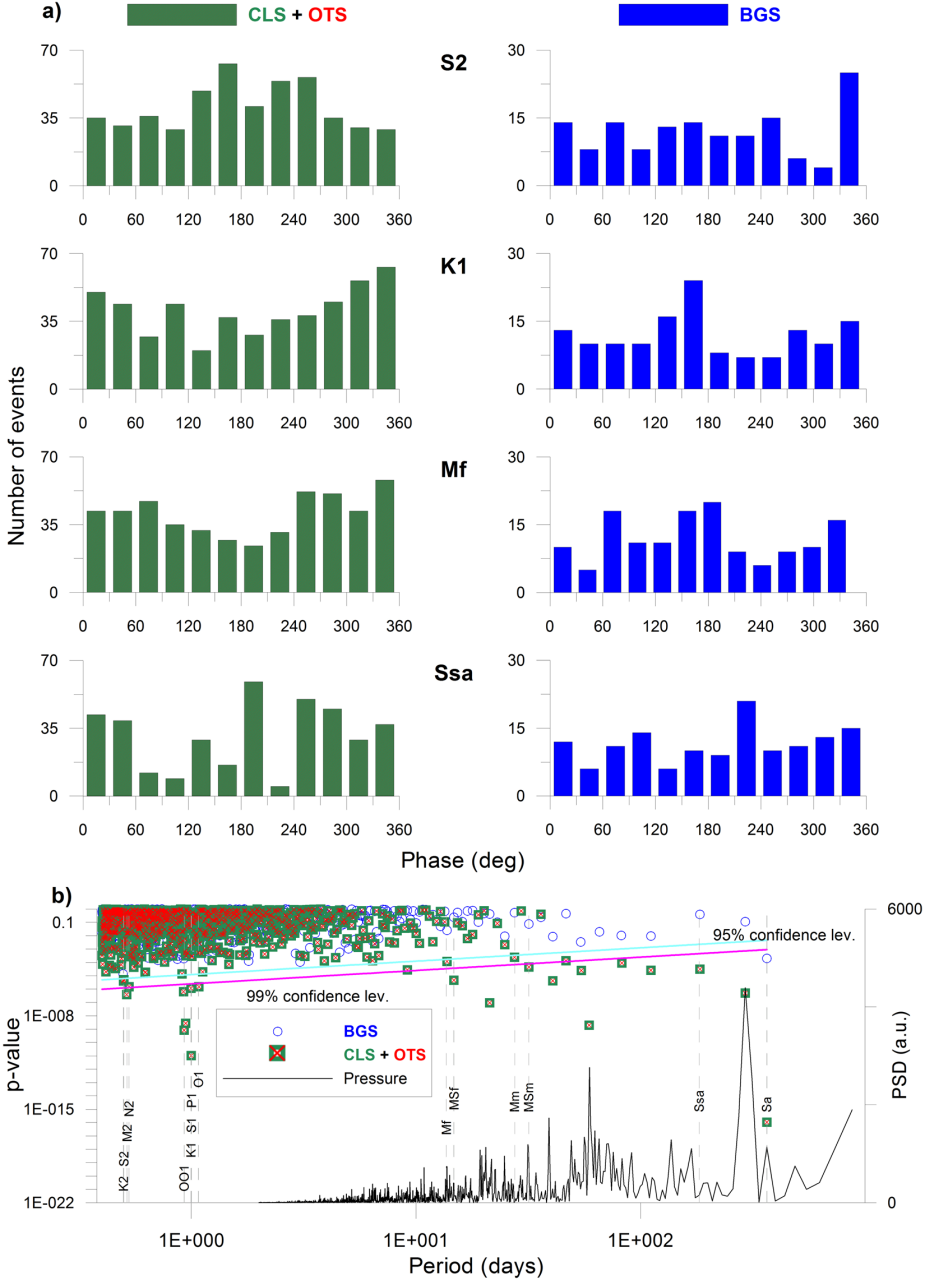
(**a**) Examples of frequency distribution of phase angles for some of the periodicities corresponding to tidal constituents; earthquakes are gathered into phase angle bins of 30° width. Results for both CLS + OTS and BGS seismic catalogues are shown; (**b**) Schuster spectrum over the CLS + OTS and BGS catalogues, for a set of periods between 0.4 and 366 days. *p*-values exceeding the 95% confidence level (cyan line) are considered significative of non-random distribution; magenta line corresponds to the 99% confidence level. The dashed vertical lines mark the main tidal periods. The power spectral density (PSD) of the atmospheric pressure relative to 2005–2016 time interval is also reported (black line).

As further step, in order to evaluate which periods related to different natural phenomena match the occurrence of the VTs, we performed a statistical analysis on the seismic catalogues by applying the HiCum approach^22^ combined with the Schuster test^23^ (see Method section for details). This test assumes that earthquakes are time-independent events, therefore it could be biased by the presence of seismic swarms in the CLS + OTS catalogue. On the other hand, the swarms are an important component in the CFc seismicity and discarding them would be equivalent to remove a significant part of the signals we want to test. To solve this issue, we used an approach aimed at removing in the seismic catalogue the time-dependent events which can yield to false periodicities, but contemporary, preserving the timing information related to the occurrence of the single swarm. Worldwide the seismic swarms show a variety of patterns depending on their source mechanism and on the environment (tectonic, volcanic) and there is no a universal rule to identify them; however, some criteria can be derived by observing their characteristics^24^. Fortunately, the CFc swarms nearly maintained similar pattern over the last 18 years (see the beginning of this Section), so it is not difficult to individuate them in the seismic catalogue by means of statistical tools. After performing an inter-event time (IET) analysis on the 2005–2016 CFc seismicity, we found a bi-modal distribution which corresponds to two ensembles divided by a time-interval of about 1 day. As also pointed out by Chiodini *et al*.^16^, below the 1-day threshold the population mainly consists of swarm events. Moreover, our statistical analysis on this population evidences that there is the 87% of probability to observe IET < 30 min, which therefore represents a reasonable threshold to identify a swarm. On this basis, we chose as criterion for the swarm detection, the occurrence of a sequence of at least 10 earthquakes with IET < 30 min. Once the swarms were identified in the CLS + OTS catalogue, we retained only the maximum magnitude VT belonging to that swarm^25^, which is considered somewhat as the “marker” of the activity. In this way, the swarm timing is preserved and the exclusion of the other events separated only by a small time fraction prevents from biasing the results of the Schuster test. The “de-swarmed” CLS + OTS, the BGS and the combination BGS + CLS + OTS catalogues were then tested for a continuous set of periodicities ranging between 0.4 and 366 days, thus encompassing the periods which corresponds to those of both the main tidal constituents, as well as other factors such as atmospheric pressure and temperature cycles (Table 2, Fig. 6a). The retrieved Shuster spectrum estimated on the “de-swarmed” CLS + OTS catalogue is shown in Fig. 6b; *p*-values exceeding the 95% confidence level indicate that the events do not occur randomly in time. Significative *p*-values correspond to the periods of the main tidal constituents (Table 2), some of them even exceeding the 99% confidence level; at longer time scales (>1 day) low *p*-values (meaning high probability) also match the spectral peaks of the atmospheric pressure spectrum. It is noteworthy that the role of the atmospheric pressure in modulating the ground deformation with a nearly 18-day periodicity has also been evidenced in a recent study on borehole tiltmetric time series at the CFc^26^. The Schuster test inferences also hold for the CLS catalogue minus the OTS, and for the catalogue composed only by the OTS. Moreover, the statistical test leads to the rejection of the null hypothesis (e.g. that earthquakes occur randomly) both including or excluding the September 2012 swarm (as well as the whole year 2012) in the seismic catalogue, or limiting the analysis to the sub-interval 2005–2011, 2005–2012 and 2013–2016.

**Table 2:** Main periodicity related to tidal and atmospheric phenomena.

Period (day)	Origin
0.4986	Lunisolar semidiurnal K2
0.5	Principal solar semidiurnal S2, atmospheric pressure
0.5175	Principal lunar semidiurnal M2
0.5274	Larger lunar elliptic semidiurnal N2
0.9294	Lunar diurnal OO1
0.9973	Lunisolar diurnal K1
1	Principal solar diurnal S1, air temperature
1.0028	Principal solar declination diurnal P1
1.0758	Principal lunar declination diurnal O1
13.661	Lunisolar fortnightly Mf
14.765	Lunisolar synodic fortnightly MSf
27.555	Lunar monthly Mm
31.812	Solar monthly MSm
182.621	Solar semiannual Ssa
365.260	Solar annual Sa

On the contrary, the occurrence frequency of the BGS is not significantly correlated with the tested periods: the Schuster *p*-value (Fig. 6b) never exceeds the 95% confidence level, except for the solar annual Sa. However for this tidal period, the retrieved *p*-value indicate weaker correlation compared to that of the CLS + OTS. Finally, for the combination BGS + CLS + OTS, the dataset still results well correlated with the periods corresponding to the main tidal constituents and to the atmospheric pressure variations, although at a lower degree compared with the sole CLS + OTS; this was expected because the BGS represents a percentage less than 23% of the de-clustered catalogue.

## Discussion

Our data analysis clearly indicates that the occurrence of clustered seismicity (CLS + OTS) at the CFc has a cyclic behaviour on several time scales, from semidiurnal and diurnal to longer periods, such as fortnightly, monthly, semiannual and annual. The retrieved periods match those of the main lunar and solar tidal constituents, although the S1 and S2 (exactly 24-h and 12-h periodicities) can be ascribed not only to the Earth tides but also to other effects such as temperature and atmospheric pressure variations. Influence of the atmospheric pressure at longer time scales also appears. Moreover, a clear dependence on rainfall arises from the analysis of the hydrological parameters, indicating a strong seasonal periodicity until the September 2012 swarm, with much more earthquakes occurring during the wet than in the dry season; after that swarm, earthquake yearly distribution has become more homogeneous, with a slightly higher number of events during the dry season. The other relevant fact is that, for the entire observational interval (2005–16), and with the sole exception of 2016, the most numerous (and energetic) swarm of each year has occurred in the wet season. These observations evidence that clustered seismicity at the CFc has been strongly influenced by non-magmatic triggers, as tidal and/or hydrological cycles, almost until the September 2012 swarm, after which external triggers have still been active but at a lesser extent.

What are the implications of these facts for the evaluation of the activity state of the CFc and the elaboration of medium-term volcanic risk scenarios? Looking at the previous related literature^16^, VT seismicity, ground deformation and concentration of the most temperature-sensitive species in fumarolic gases are quasi-synchronous proxies of the temperature-pressure state of the 2 km-deep hydrothermal aquifer of the CFc, e.g. of the unrest level of that caldera. Chiodini *et al*.^16^ demonstrated that recurrent increases of VT earthquakes with high inter-arrival times are compatible with injections of magmatic fluids in the hydrothermal aquifer. But, for better addressing the role of these fluid injection episodes for the reconstruction of the activity state of the CFc, we should answer to the question whether these injections are related to active or passive magmatic degassing. Active degassing implies exsolution of volatiles from an ascending magma re-equilibrating to lower confining pressure conditions^27^. Passive degassing is from a magma standing at a certain depth and losing volatiles by diffusion and/or exsolution due to oversaturation, which can be reached through melt volume reduction due to progressive crystallization^28^ and/or depressurization induced by changes in the physical state of the caprock. Different degassing modalities imply different theoretical volcanic risk scenarios. The ascent of magma (active degassing) could eventually lead to a magmatic or phreato-magmatic eruption, passive degassing could drive the hydrothermal system beyond critical pressure conditions, giving rise to a phreatic eruption when the lithostatic pressure is overwhelmed.

The magma ascent scenario is seemingly assignable to the September 2012 swarm (and the related fluid injection), whose characters are compatible with a shallow dyke intrusion, revealed by the huge increase in the ground uplift rate (the main observed during the present unrest), and the anomalous hypocentral locations, falling outside the well confined volume inside which VT seismicity is usually generated. The other modelled injections can be reasonably referred to the migration of fluids without involvement of magma movements, e.g. to increments in passive degassing frequently associated with VT seismicity and variations of ground uplift rates.

A simple, comprehensive model linking magmatic degassing, ground deformation and seismicity can be found in considering the magmatic-hydrothermal fluid circulation system as a cascade hydraulic circuit, where in/out fluid flow and pressure condition of each segment are regulated by those of the contiguous (below and above) ones. We first consider the active degassing case: once magmatic volatiles, progressively released by the ascending magma, have reached the hydrothermal aquifer, they cause an increment of its pressure. If the overpressure is not sufficiently compensated by a corresponding increment in concentrated and diffuse soil degassing, it will trigger ground uplift (aseismic deformation) and/or crustal stress accumulation, eventually evolving in VT seismicity generation. Conversely, in the passive degassing case, we can explain fluid injections considering an opposite event chain (from the surface of the Earth toward its interior), without losing the physical consistency of the degassing model. Earth tides, variations of the water content in the shallow crust and of the atmospheric pressure generate forces, whose moduli and versa change periodically from centrifugal to centripetal, interfering in phase addition or opposition with the crustal vertical displacement, modulating the opening or closure of voids (both fractures and porosity). The combined action of these processes influences shallow crustal permeability. For example, strain-induced permeability increases in volcanic rocks have been evidenced by Farquharson *et al*.^29^. In addition, as recently shown by a laboratory study on the CFc tuff samples, pressure increases cause a reduction of the permeability while thermal stresses due to temperature rise induce an increment^30^. In turn, permeability changes critically affect the state of the system acting as regulator of the internal pressure^31^: variations of the permeability thus cyclically foster or oppose to hydrothermal degassing. If outgassing is opposed, we will have a pressurization of the hydrothermal aquifer, which will increment the stress field favouring ground deformation and/or seismicity. On the contrary, hydrothermal gas outflow will increase during phases of increasing permeability, causing a depressurization of the aquifer, which will claim more volatiles from the deeper segments of the magmatic supply system, causing in turn a magmatic fluid injection configured as “passive degassing”.

It is noteworthy that passive degassing, triggered by exogenous processes, could in turn promote vertical magma movements (and consequent active degassing). Magma ascent is basically due to buoyancy, driven by the negative density differential between a magma batch and the surrounding melt, and magma density is influenced by the amount of free gases circulating inside it. As a consequence, a depressurization chain, starting from the surface of the Earth and progressively propagating down to the deeper segments of the magmatic supply system, can foster the exolution of gases in a deep magmatic body, diminishing its density and driving its ascent^32^.

Besides, exogenous triggers of volcanic eruptions are not a novelty: seasonality of eruption rates has been attributed to the crustal load related to variations in the atmospheric pressure and/or movement of surface water mass during the annual hydrological cycle^33–35^. The role of rainfall in modulating internal processes has well been documented at Piton de la Fournaise and Montserrat volcanoes^36,37^. If the crust is near a critical state, pore pressure and permeability changes induced in the rocks by hydrological cycles can trigger earthquakes as poroelastic response to the surface water flux^38,39^. Another effect of rain infiltration is the diversion of the soil gas flux from porosity to fractures because, due to their minor dimension, pores reach the water saturation condition (that blocks the flux of gases) earlier than fractures^40,41^; the final effect is a reduction of the outgassing capability of the shallow crust, which in turn triggers a pressure increment of hydrothermal systems, especially if hosted in fractured zones.

Finally, many researchers suggest that the Earth tidal stress field acts as a possible global-scale trigger for volcanic eruptions^33,42,43^. As example, recent studies at Stromboli, Ruapehu and Axial Seamount volcanoes evidence how tidal cycles can control the timing of the eruptions^44–46^. It is noteworthy that a common outcome of all these studies is that the more the system is in prone to critical state, the more the external trigger will be effective, irrespective of its origin (rain, pressure, tides). Monitoring the time variations of these exogenous phenomena and their correlation level with the internal seismic/volcanic activity is therefore useful to assess risk scenarios.

The abovementioned considerations give new insights about the recent dynamic of the CFc, suggesting that the recurrent recrudescence of ground deformation episodes, VT clustered seismicity and geochemical anomalies in the hydrothermal aquifer are the result of the combined action between endogenous and exogenous processes. Obviously, exogenous processes cannot be the primary cause of huge changes affecting a magmatic system: the much less energetic forces generated by tidal or hydrological cycles cannot reactivate a dormant volcano. However, if the shallower segment of a volcanic system is close to an instability condition, exogenous processes can supply the “energetic differential” that will trigger its evolution, including dyke intrusions, phreatic or volcanic eruptions. Our results suggest that a complex mixing of endogenous and exogenous phenomena takes place at the CFc, and that its monitoring should not be focused only to the surficial indicators of deep processes, but it should also encompass the study of those exogenous mechanisms potentially able to trigger volcanic activity. In doing this, particular care must be taken to the intrinsic chaotic character of these complex phenomena: the deep part of a volcanic system evolves independently of the dynamics governing exogenous processes, and the resulting interactions are far from following a simple, deterministic pathway.

## Methods

### Seismic Data Acquisition

The seismicity of the CFc is monitored by the permanent and the mobile seismic networks of INGV-OV (black triangles in Fig. 1). The dataset used in this paper spans the observational interval from 2005 to 2016, during which the seismic networks have undergone to several technological improvements. Currently, the permanent network comprises 23 stations, 18 of which are digital dataloggers equipped with broadband three-component Guralp CMG40T, Guralp 3TB/5TB or Trillium 120 P seismometers, and 5 are analogical stations, with short-period 1 Hz Mark L4C, 1 Hz Lennartz LE-3Dlite, or Geotech S13 sensors. The signals are continuously acquired at a sampling rate of 100 Hz and telemetered to the acquisition centre in Napoli. The mobile network is composed of 15 stand-alone dataloggers, equipped with three-component 1 Hz Lennartz LE-3Dlite, Lennartz LE3D/20 s, Geotech KS2000 or Guralp CMG-40T seismometers. The data are locally stored at sampling rate of 125 Hz or 100 Hz.

The arrival times of the VT events, recorded by the two networks, are routinely picked and integrated. For the analysed observational interval, we used the integrated pickings to perform a 3D probabilistic non-linear location by using the NLLOC software^47^. To search for the best solution over a 3D grid, the algorithm calculates the theoretical travel times at the different stations in a 3D velocity structure derived from the SERAPIS tomography model of Judenherc & Zollo^48^. The results are reported in Fig. 1.

### Normal probability plot technique

The Normal Probability Plot method^17,18^ is a graphical approach to visually verify if a discrete small dataset follows an hypothesized statistical distribution. The sorted data are plotted against approximated value of the means or medians following the hypothesised statistics, such as the cumulative percentage of the data. In general the procedure begins by sorting the observations from the smallest to the largest, x(1), x(2), …, x(n), and then the x(i) are plotted against their relative cumulative frequency

2 $${f}_{i}=\frac{i}{n+1};$$

with the y-axis scaled for the established distribution. The largest data point will not corresponds to the 100% frequency, allowing for future observations to be larger. Symmetrically for the smallest. If the distribution effectively describes the observations, they fall approximately along a straight line. Often Gaussian distribution is assumed and the data distribution is evaluated in a percent semi-log plot.

In general a subjective visual interpretation is given, although it is possible to provide statistical evaluations^17^. To check the sorted data (VT m^−1^) distribution we followed a statistical approach. We used an ensemble of probability functions (linear, logarithmic, exponential, power law, polynomial and orthogonal polynomial) to fit the data cumulative frequencies as the number of data points (n) varies, from n = 3 to the total number of observations (n = 144). For each probability function, the RMS (Root Mean Square) parameter was estimated (where possible) as function of n. All the obtained RMS functions were then matched to identify the bounds of seismicity populations, the corresponding best representative probability functions and the outliers.

### Statistical tests HiCum and Shuster

HiCum (Histogram Cumulating) is an analysis method^22^ to detect periodicity of a time series, by transforming and stacking time based observations into periodical based data. In particular, it is applied to investigate the correlation between the tidal potential components and earthquake occurrences^49,50^. The phase angle, *α*_*i*_, of the time occurrence of each earthquake is obtained by equation (3):

3 $${\alpha }_{i}=[(\frac{{t}_{i}-{T}_{0}}{T})]-int[(\frac{{t}_{i}-{T}_{0}}{T})]\times 360;$$

where *t*_*i*_ is the time of occurrence of the *i*-th earthquake *T*_0_ is the reference time fixed from astronomical data and *T* is the period of the considered tidal component. For each selected tidal period T, a histogram of the *α*_*i*_ is obtained by dividing the 0–360° interval into M bins with length equal to 360°/M. Therefore, each phase angle corresponding to the events of the seismic catalogue is stacked in the proper bin.

The correlation between the tidal components and earthquake occurence is investigated by using the Shuster test^22,51^. Each earthquake corresponds to a unit length vector in the direction of its tidal phase angle *α*_*i*_. The vectorial sum D is defined by equation (4):

4 $${\rm{D}}=\,{(\sum _{{\boldsymbol{i}}=1}^{{\boldsymbol{N}}}cos{{\boldsymbol{\alpha }}}_{{\boldsymbol{i}}})}^{2}+{(\sum _{{\boldsymbol{i}}=1}^{{\boldsymbol{N}}}sin{{\boldsymbol{\alpha }}}_{{\boldsymbol{i}}})}^{2};$$

where *N* is the number of earthquakes. When *α*_*i*_ is distributed randomly, the probability, *p*, that the length of a vectorial sum is equal to or larger than D is given by equation (5):

5 $$p={\bf{e}}{\bf{x}}{\bf{p}}(-\frac{{D}^{2}}{N});$$

*p* represents the significance level for rejecting the null hypothesis that earthquakes occur randomly with respect to the tidal phase: thus the smaller the *p*, the greater the correlation between the Earth tide and earthquake occurrence. Generally a threshold of 5% is adopted: probabilities *p* < 5% are considered non-random and the correlation is judged significant, while *p*-values greater than 5% correspond to random event distribution.

The Schuster test alone does not establish a sufficient condition to assert the periodicity in an earthquake catalogue^52,53^. Indeed the detected non-uniformity at the period *T* can also be induced by bursts of seismicity such as aftershock sequences or seismic swarms. An extension of the technique, which consists in computing a spectrum of Schuster *p*-values over a continuous a range of periods, is therefore recommended when dealing with seismic catalogues. In this case, the detection level is period-dependent; for example, a probability threshold of 5% at the period *T* is defined according to the relationship:

6 $$\delta =0.05\times \frac{T}{t};$$

where *t* is the total observation interval, corresponding to the catalogue duration.

### Meteo-hydrological data

**Figure 7:**
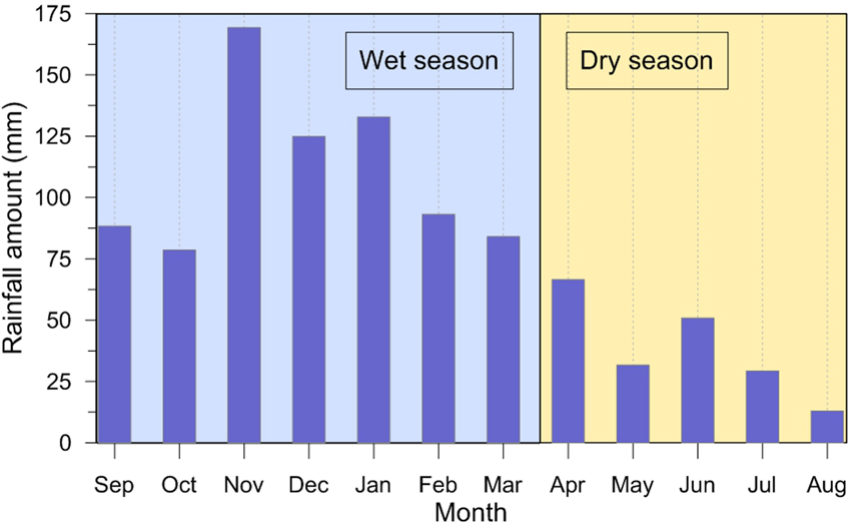
Monthly rainfall amounts (2005–2016 average) and subdivision of the average hydrological year at the CFc.

Rainfall amounts refer to a synthetic hydrological series built on data mainly acquired in the Pozzuoli station of the Campania Regional Agrometeorological Centre network, and completed for the missing periods with values from the closest (both in terms of distance and compatible orographic conditions) available stations (both from the same and other publicly available networks). Based on the monthly averages of rainfall amounts for the time span 2005–2016 (see Table of data) we divided the hydrological average year into two sub-periods (Fig. 7): the wet season, from September to March, and the dry season, from April to August. Atmospheric pressure data are from LIRN station of the Wunderground weather network.

## Electronic supplementary material

Dataset 1

## Data Availability

Seismic, Earth tide and rainfall data available in the Excel spreadsheet annexed as online supplementary material of this article. Ground deformation data available at the doi 10.4401/ag-6431 and at the http://www.ov.ingv.it/ov/it/bollettini.html. Atmospheric pressure data available at the https://www.wunderground.com/history/airport/LIRN.
